# Geometry-invariant abnormality detection

**DOI:** 10.1007/978-3-031-43907-0_29

**Published:** 2023-01-10

**Authors:** Ashay Patel, Petru-Daniel Tudosiu, Walter Hugo Lopez Pinaya, Olusola Adeleke, Gary Cook, Vicky Goh, Sebastien Ourselin, M. Jorge Cardoso

**Affiliations:** King’s College London, London, WC2R 2LS, United Kingdom

## Abstract

Cancer is a highly heterogeneous condition best visualised in positron emission tomography. Due to this heterogeneity, a general-purpose cancer detection model can be built using unsupervised learning anomaly detection models. While prior work in this field has showcased the efficacy of abnormality detection methods (e.g. Transformer-based), these have shown significant vulnerabilities to differences in data geometry. Changes in image resolution or observed field of view can result in inaccurate predictions, even with significant data pre-processing and augmentation. We propose a new spatial conditioning mechanism that enables models to adapt and learn from varying data geometries, and apply it to a state-of-the-art Vector-Quantized Variational Autoencoder + Transformer abnormality detection model. We showcase that this spatial conditioning mechanism statistically-significantly improves model performance on whole-body data compared to the same model without conditioning, while allowing the model to perform inference at varying data geometries.

## 1 Introduction

The use of machine learning for anomaly detection in medical imaging analysis has gained a great deal of traction over previous years. Most recent approaches have focused on improvements in performance rather than flexibility, thus limiting approaches to specific input types – little research has been carried out to generate models unhindered by variations in data geometries. Often, research assumes certain similarities in data acquisition parameters, from image dimensions to voxel dimensions and fields-of-view (FOV). These restrictions are then carried forward during inference [26, 5]. This strong assumption can often be complex to maintain in the real-world and although image pre-processing steps can mitigate some of this complexity, test error often largely increases as new data variations arise. This can include variances in scanner quality and resolution, in addition to the FOV selected during patient scans. Usually training data, especially when acquired from differing sources, undergoes significant pre-processing such that data showcases the same FOV and has the same input dimensions, e.g. by registering data to a population atlas. Whilst making the model design simpler, these pre-processing approaches can result in poor generalisation in addition to adding significant pre-processing times [27, 12, 14]. Given this, the task of generating an anomaly detection model that works on inputs with a varying resolution, dimension and FOV is a topic of importance and the main focus of this research.

Unsupervised methods have become an increasingly prominent field for automatic anomaly detection by eliminating the necessity of acquiring accurately labelled data [7, 4] therefore relaxing the stringent data requirements of medical imaging. This approach consists of training generative models on healthy data, and defining anomalies as deviations from the defined model of normality during inference. Until recently, the variational autoencoder (VAE) and its variants held the state-of-the-art for the unsupervised approach. However, novel unsupervised anomaly detectors based on autoregressive Transformers coupled with Vector-Quantized Variational Autoencoders (VQ-VAE) have overcome issues associated with autoencoder-only methods [22, 23]. In [23], the authors explore the advantage of tractably maximizing the likelihood of the normal data to model the long-range dependencies of the training data. The work in [22] takes this method a step further through multiple samplings from the Transformer to generate a non-parametric Kernel Density Estimation (KDE) anomaly map.

Even though these methods are state-of-the-art, they have stringent data requirements, such as having a consistent geometry of the input data, *e.g*., in a whole-body imaging scenario, it is not possible to crop a region of interest and feed it to the algorithm, as this cropped region will be wrongly detected as an anomaly. This would happen even in the case that a scan’s original FOV was restricted [18].

**Fig. 1:**
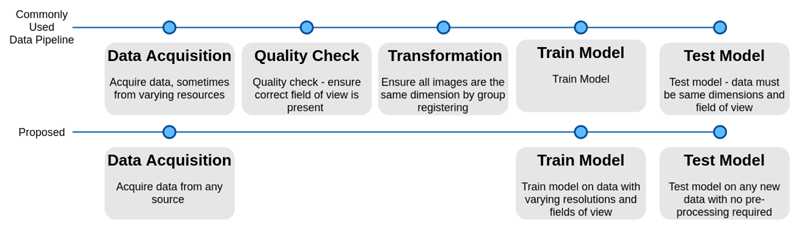
Flowchart showcasing traditional data pipelines for developing machine learning models in medical imaging (top) vs. the reduced pipeline for our approach (bottom)

As such, we propose a geometric-invariant approach to anomaly detection, and apply it to cancer detection in whole-body PET via an unsupervised anomaly detection method with minimal spatial labelling. Through adapting the VQ-VAE Transformer approach in [22], we showcase that we can train our model on data with varying fields of view, orientations and resolutions by adding spatial conditioning in both the VQ-VAE and Transformer. Furthermore, we show that the performance of our model with spatial conditioning is at least equivalent to, and sometimes better, than a model trained on whole-body data in all testing scenarios, with the added flexibility of a “one model fits all data” approach. We greatly reduce the pre-processing requirements for generating a model (as visualised in Figure 1), demonstrating the potential use cases of our model in more flexible environments with no compromises on performance.

## 2 Background

The main building blocks behind the proposed method are introduced below. Specifically, a VQ-VAE plus a Transformer are jointly used to learn the probability density function of 3D PET images as explored in prior research [22, 23, 25].

### 2.1 Vector-Quantized Variational Autoencoder

The VQ-VAE model provides a data-efficient encoding mechanism — enabling 3D inputs at their original resolution — while generating a discrete latent representation that can trivially be learned by a Transformer network [21]. The VQ-VAE is composed of an encoder that maps an image *X* ∈ ℝ^*H×W ×D*^ onto a compressed latent representation $Z\in {\mathbb{R}}^{h\times w\times d\times n_{z}}$ where *n*_*z*_ is the latent embedding vector dimension. *Z* is then passed through a quantization block where each feature column vector is mapped to its nearest codebook vector. Each spatial code $Z_{ijl}\in {\mathbb{R}}^{n_{z}}$ is then replaced by its nearest codebook element $e_{k}\in {\mathbb{R}}^{n_{z}}$, *k* ∈ 1, …, *K* where *K* denotes the codebook vocabulary size, thus obtaining *Z*_*q*_. Given *Z*_*q*_, the VQ-VAE decoder then reconstructs the observations $\hat{X}\in {\mathbb{R}}^{H\times W\times D}$. The architecture used for the VQ-VAE model used an encoder consisting of three downsampling layers that contain a convolution with stride 2 and kernel size 4 followed by a ReLU activation and 3 residual blocks. Each residual block consists of a kernel of size 3, followed by a ReLU activation, a convolution of kernel size 1 and another ReLU activation. Similar to the encoder, the decoder has 3 layers of 3 residual blocks, each followed by a transposed convolutional layer with stride 2 and kernel size 4. Finally, before the last transposed convolutional layer, a Dropout layer with a probability of 0.05 is added. The VQ-VAE codebook used had 256 atomic elements (vocabulary size), each of length 128. The CT VQ-VAE was identical in hyperparameters except each codebook vector has length 64. See Appendix A for implementation details.

### 2.2 Transformer

After training a VQ-VAE model, the next stage is to learn the probability density function of the discrete latent representations. Using the VQ-VAE, we can obtain a discrete representation of the latent space by replacing the codebook elements in *Z*_*q*_ with their respective indices in the codebook yielding *Z*_*iq*_. To model the imaging data, we require the discretized latent space *Z*_*iq*_ to take the form of a 1D sequence *s*, which we achieve via a raster scan of the latent. The Transformer is then trained to maximize the log-likelihoods of the latent tokens sequence in an autoregressive manner. By doing this, the Transformer can learn the codebook distribution for position *i* within *s* with respect to previous codes *p*(*s*_*i*_) = *p*(*s*_*i*_|*s*_*<i*_). As with [22], we additionally use CT data to condition the Transformer via cross-attention using a separate VQ-VAE to encode the CT. This transforms the problem to learning the codebook distribution at position *i* as *p*(*s*_*i*_) = *p*(*s*_*i*_|*s*_*<i*_, *c*) where *c* is the entire CT latent sequence. The performer used in this work corresponds to a decoder Transformer architecture with 14 layers, each with 8 heads, and an embedding dimension of 256. Similarly the embedding dimension for the CT data and the spatial conditioning data had an embedding dimension of 256. See Appendix B for implementation details.

### 2.3 Anomaly Detection via Kernel Density Estimation Maps

Building on [22], given a sample for inference, a tokenized representation *Z*_*iq*_ is extracted from the VQ-VAE. Then, the representation is flattened into *s* where the trained Transformer model obtains the likelihoods for each token. These inferred likelihoods represent the probability of each token appearing at a certain position in the sequence - *p*(*s*_*i*_) = *p*(*s*_*i*_|*s*_*<i*_, *c*). This can then be used to single out tokens with low probability, i.e. anomalous tokens. We then resample anomalous tokens *p*(*s*_*i*_) *< t* where *t* is the resampling threshold chosen empirically using the validation set performance. Anomalous tokens are then replaced with higher likelihood (normal) tokens by resampling from the Transformer. We can then reshape the “healed” sequence back into its 3D quantized representation to feed into the VQ-VAE to generate a healed reconstruction *X*_*r*_ without anomalies.

In this work, abnormalities are defined as deviations between the distribution of “healed” reconstructions and the observed data, measured using a Kernel Density Estimation (KDE) approach. We generate multiple healed latent sequences by sampling multiple times for each position *i* with a likelihood *p*(*s*_*i*_) <* t*. In each resampling, the Transformer outputs the likelihood for every possible token at position *i*. Based on these probabilities, we can create a multinomial distribution showcasing the probability of each token. We can then randomly sample multiple tokens. Each of these healed latent spaces is then decoded via the VQ-VAE multiple times with dropout. This generates multiple healed representations of the original image. A voxel-wise KDE anomaly map is generated by fitting a KDE independently at each voxel position to estimate the probability density function *f* across reconstructions. This is then scored at the original intensity of that voxel in the scan. Our KDE implementation used 60 samples for each anomalous token in *s*, followed by five decodings with dropout, yielding 300 “healed” reconstructions that are then used to calculate the KDE.

## 3 Method

### 3.1 VQ-VAE Spatial Conditioning

To date, there has been little research on generating autoencoder models capable of using images of varying sizes and resolutions (i.e. the input tensor shape to a autoencoder is assumed to be fixed). Although fully convolutional models can ingest images of varying dimensions, we have found that using training data with varying resolutions resulted in poor auto-encoder reconstructions. In this work, we take inspiration from CoordConv [20] as a mechanism to account for some level of spatial awareness, an approach which has been applied to various tasks in medical imaging scenarios with ranging levels of success [19, 1].

A CoordConv layer is a concatenation of channels to the input image referencing a predefined coordinate system. After concatenation, the input is simply fed through a standard convolutional layer. For a 3D scan, we would have 3 coordinates, *ijk*, where the *i* coordinate channel is an *h* × *w* × *d* rank-1 matrix with its first row filled with 0’s, its second row with 1’s, and so on. This would be the same for the *j* coordinate channel, except the columns would be filled with constant values, not the rows, and likewise for the *k* coordinate channel in a depth-wise fashion. These channels are then normalised between [0, 1].

**Fig. 2:**
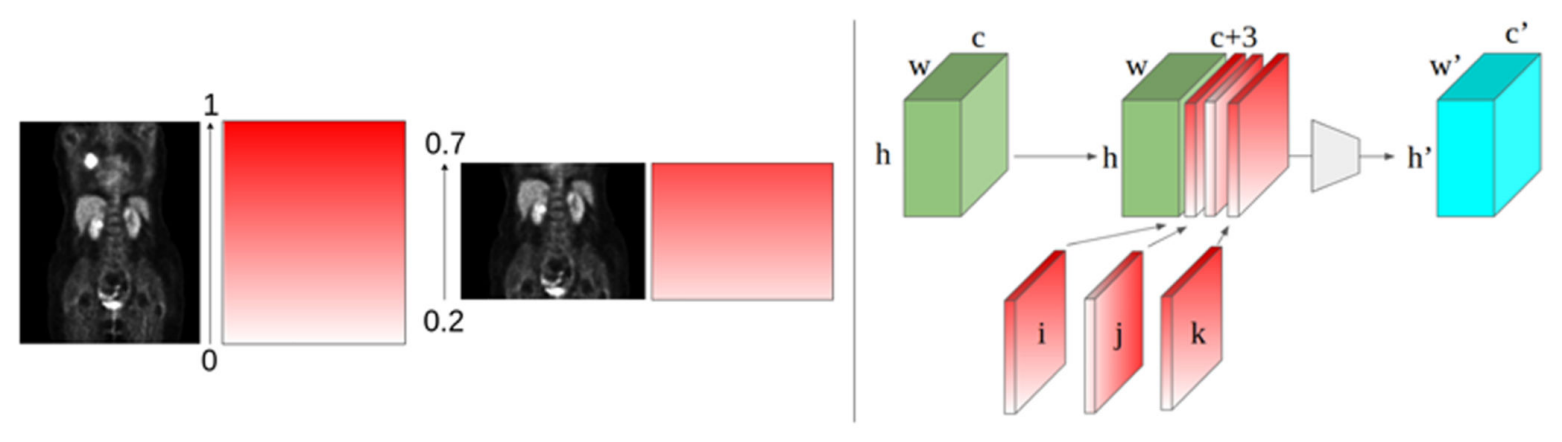
CoordConv example showing whole-body image with values from 0 to 1 vs. a cropped image with values from 0.2 to 0.7 to reflect the field of view

The advantage of the CoordConv implementation is the constant scale of 0-1 across the channels regardless of image resolution. For example, two whole-body images with large differences in voxel-size will have CoordConv channels from 0-1 along each axis, thus conveying the notion of spatial resolution to the network. We found when training the VQ-VAE model on data with varying resolutions and dimensions that reconstructions showcased unwanted and significant artifacts, while by adding the CoordConv channels this issue was not present (See Appendix C for examples). Furthermore, when dealing with images of a ranging FOV, we adapted the [0,1] channel values to convey the image’s FOV. For example, suppose a whole body image (neck to upper leg) represented our range [0,1] where 0 is the upper leg, and 1 is the neck. In that case, we can contract this range to represent the area displayed in the image (Figure 2). In doing so, we convey information about the FOV to the VQ-VAE through CoordConv layers. Note that while the proposed model assumes only translation and scale changes between samples, it can be trivially extended to a full affine mapping of the coordinate system (including rotations/shearing between samples).

We used random crops during training to simulate varying FOVs of whole-body data. The random crop parameters are then used to define the coordinate system. For the implementation of the CoordConv layer, these channels are added once to the original input image and at the beginning of the VQ-VAE decoder, concatenated to the latent space, using the same value ranges but at a lower resolution given the reduced spatial dimension of the latent space.

### 3.2 Transformer Spatial Conditioning

Numerous approaches have used Transformers in the visual domain [7, 8]. Given that Transformers work natively on 1D sequences, the spatial information in images is often lost. While various works have aimed to convey the spatial information of the original image when projected onto a 1D sequence [15, 29], we require our spatial positioning to encode both where in the image ordering a token belongs, and where the token belongs in the context of the whole body. As the images have different FOVs and the image resolution, this results in varying token sequence lengths. As such, the Transformer must be informed of the location of a given token in relationship to the whole-body.

To do this, we use the same CoordConv principle applied to the input fed to the VQ-VAE. In order to map image coordinates to the token latent representation, we apply average pooling to each CoordConv channel separately, with kernel size and stride equal to the downsampling used in the VQ-VAE (8 used in this research). This gives us three channels *i, j, k* in the range of [0,1], the same dimension as our latent space, but at lower spatial resolution to the original input. We then bin each value in each channel and combine the three values using base notation. For example, we use 20 bins (equal bins of 0.05), to which the final quantized spatial value for a given token is given as *sp*_*ijk*_ = *b*_*i*_ + *b*_*j*_ × *B* + *b*_*k*_ * *B*^2^ where *sp* is the quantized spatial value allocated to a given token at position *ijk* in the latent space, and *b* represents the binned value along a given channel for that token, and *B* is a pre-defined bin size. The choice of *B* = 20 bins was empirically chosen to closely resemble the average latent dimension of images.

**Fig. 3:**
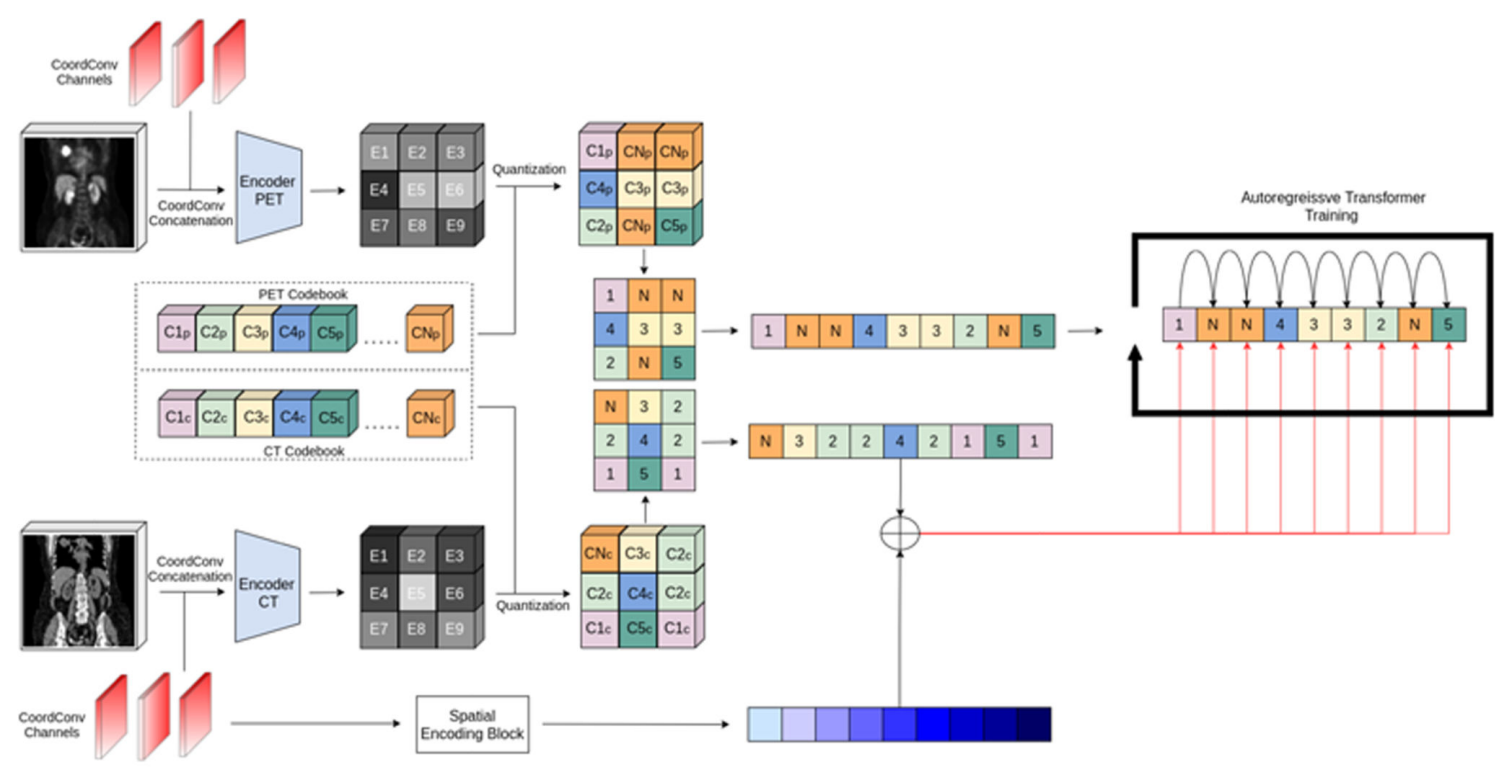
Pipeline for Transformer training. PET and CT are encoded to generate a discrete latent space. CoordConv layers are used to generate the spatial conditionings that are added to the CT conditioning and fed to the Transformer via cross-attention

During training, whole-body images and random crops are used. The spatial conditioning tokens are then generated and fed through an embedding layer of equal dimension to the CT embedding. The two embedded sequences (CT and spatial) are then added together and fed to the Transformer via cross-attention. For reference, this mechanism can be visualised in Figure 3.

### 3.3 Data

For this work we leveraged whole-body PET/CT data from different sources to explore the efficacy of our approach for varying image geometries. 211 scans from NSCLC Radiogenomics [2, 3, 17, 10] combined with 83 scans from a proprietary dataset constitute our lower resolution dataset with voxel dimensions of 3.6x3.6x3mm. From this, we split the data to give 210 training samples, 34 validation and 50 testing. Our higher resolution dataset uses AutoPET [16, 11] (1014 scans) with voxel dimensions of 2.036x2.036x3mm. From this, 850 scans are used for training, 64 for validation and 100 for testing.

All baseline models work in a single space with constant dimensions, obtained by registering the AutoPET images to the space of the NSCLC dataset.

For evaluation, we use four testing sets: a lower resolution set derived from both the NSCLC and the private dataset; a higher resolution set from AutoPET; a testing set with random crops of the same NSCLC/private testing dataset and finally a testing set that has been rotated through 90 degrees using the high resolution testing data. As the cropped and rotated dataset cannot be fed into the baseline models, we pad the images to the common image sizing before inference.

## 4 Results

The proposed model was trained on the data described in 3.3, with random crops applied while training. Model and anomaly detection hyperparameter tuning was done on our validation samples using the best DICE scores. We then test our model and baselines on 4 hold-out test sets: a low-resolution whole-body set, a low-resolution cropped set, a high-resolution rotated set and a high-resolution test set of PET images with varying cancers. The visual results shown in Figure 5 show outputs rotated back to the original orientation. We measure our models’ performance using the DICE score, obtained by thresholding the residual/density score maps. In addition, we calculate the area under the precision-recall curve (AUPRC) as a suitable measure for segmentation performance under class imbalance. We additionally showcase the performance of the classic VQ-VAE + Transformer approach trained on whole-body data only (without the proposed spatial conditioning), as well as the proposed CoordConv model trained with varying image geometries but without the transformer spatial conditioning to explicitly showcase the added contribution of both spatial conditionings. The full results are presented in Table 1 with visual examples shown in Figure 5. We can observe that the addition of spatial conditioning improves performance even against the same model without conditioning trained on whole-body data (Mann Whitney U test, *P <* 0.01 on high resolution and *P <* 0.001 on cropped data for DICE and AUPRC). For cropped data, models trained on whole-body data fail around cropping borders, as showcased in Figure 5. This is not the case for the models trained on varying geometries. Note that the VQ-VAE + Transformer trained on varying geometries still shows adequate performance, highlighting the resilience of the Transformer network to varying sequence lengths without any form of spatial conditioning. However, by adding the transformer spatial conditioning, we see improvements across all test sets (most significantly on cropped data and the rotated data *P <* 0.001) for both evaluation metrics. For the rotated data, we see little performance degradation in the conditioned model thanks to the spatial conditioning. The same model without conditioning showed much lower performance with higher false positives likely due to the model’s inability to comprehend the anatomical structures present due to the rotated orientation.

## 5 Conclusion

Detection and segmentation of anomalous regions, particularly for cancer patients, is essential for staging, treatment and intervention planning. Generally, the variation scanners and acquisition protocols can cause failures in models trained on data from single sources. In this study, we proposed a system for anomaly detection that is robust to variances in geometry. Not only does the proposed model showcase strong and statistically-significant performance improvements on varying image resolutions and FOV, but also on whole-body data. Through this, we demonstrate that one can improve the adaptability and flexibility to varying data geometries while also improving performance. Such flexibility also increases the pool of potential training data, as they dont require the same FOV. We hope this work serves as a foundation for further exploration into geometry-invariant deep-learning methods for medical-imaging.

## Supplementary Material

Appendix

## Figures and Tables

**Fig. 4:**
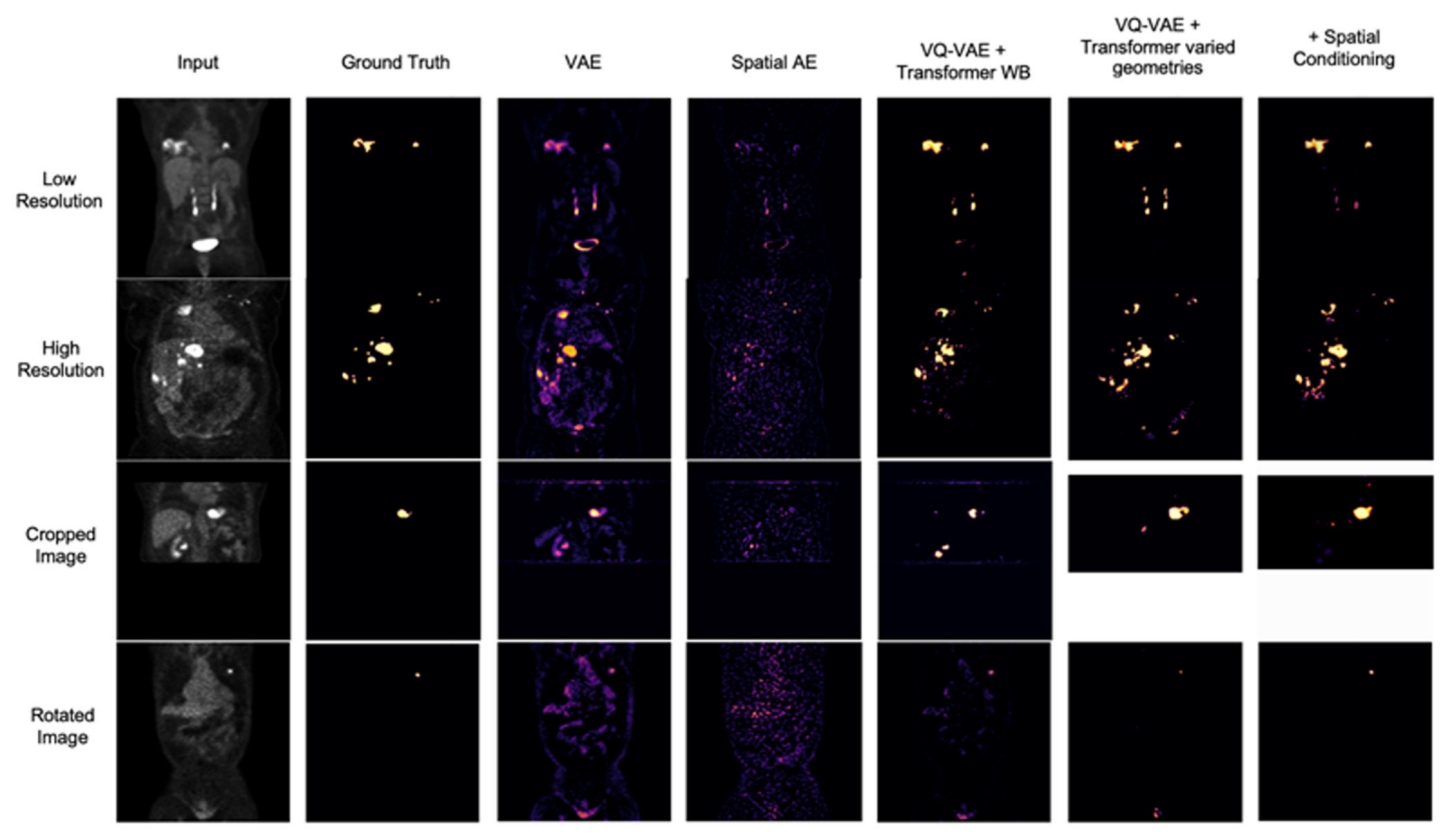
Columns display (1st) the input image; (2nd) the gold standard segmentation; (3rd) residual for the VAE, (4th) AE Spatial, (5th) a KDE anomaly map for VQ-VAE Transformer trained on the whole body, (6th) trained with varied geometries, (7th) with spatial conditioning. Results are provided for a random subject in each test set.

**Table 1:** Anomaly detection results with best achievable DICE-score (⌈*DICE*⌉) and AUPRC on test sets. Bold values indicate best performing model with underlined values showcasing statistically significant results to the next best alternative *P <* 0.05

Model		⌈*DICE*⌉			AUPRC			
**Whole Body**	Low Res	High Res	Cropped	Rotated	Low Res	High Res	Cropped	Rotated
AE Dense [4]	0.22 ±0.15	0.25 ±0.17	0.30 ±0.19	0.25 ±0.19	0.18 ±0.12	0.26 ±0.16	0.23 ±0.14	0.23 ±0.13
AE Spatial [4]	0.32 ±0.13	0.48 ±0.21	0.34 ±0.16	0.14 ±0.08	0.26 ±0.12	0.45 ±0.20	0.33 ±0.14	0.10 ±0.07
AE SSIM [6]	0.28 ±0.16	0.30 ±0.19	0.27 ±0.17	0.18 ±0.07	0.20 ±0.15	0.26 ±0.18	0.21 ±0.12	0.15 ±0.09
VAE [4]	0.35 ±0.19	0.48 ±0.22	0.34 ±0.21	0.19 ±0.08	0.33 ±0.18	0.45 ±0.20	0.35 ±0.17	0.18 ±0.09
F-Anogan [24]	0.30 ±0.18	0.42 ±0.19	0.31 ±0.15	0.20 ±0.11	0.26 ±0.15	0.40 ±0.21	0.31 ±0.18	0.19 ±0.09
VQ-VAE + Transformer [22]	0.57 ±0.07	0.65 ±0.10	0.59 ±0.10	0.31 ±0.16	0.55 ±0.09	0.64 ±0.11	0.57 ±0.10	0.29 ±0.13
**Geometry-Invariant** **(proposed)**								
VQ-VAE CoordConv	0.57 ±0.09	0.65 ±0.08	0.63 ±0.12	0.32 ±0.17	0.55 ±0.09	0.64 ±0.09	0.61 ±0.13	0.30 ±0.15
Full CoordConv	**0.58** ±0.08	**0.68** ±0.10	**0.67** ±0.10	**0.65** ±0.12	**0.56** ±0.09	**0.66** ±0.11	**0.64** ±0.11	**0.62** ±0.12

## References

[1] An CH, Lee JS, Jang JS, Choi HC (2022). Part affinity fields and coordconv for detecting landmarks of lumbar vertebrae and sacrum in x-ray images. Sensors.

[2] Bakr S, Gevaert O, Echegaray S, Ayers K, Zhou M, Shafiq M, Zheng H, Zhang W, Leung A, Kadoch M, Shrager J (2017). Data for nsclc radiogenomics collection.

[3] Bakr S, Gevaert O, Echegaray S, Ayers K, Zhou M, Shafiq M, Zheng H, Benson JA, Zhang W, Leung ANC, Kadoch M (2018). A radiogenomic dataset of non-small cell lung cancer. Scientific Data.

[4] Baur C, Denner S, Wiestler B, Albarqouni S, Navab N (2020). Autoencoders for unsupervised anomaly segmentation in brain mr images: A comparative study.

[5] Ben-David S, Blitzer J, Crammer K, Pereira F (2006). Analysis of representations for domain adaptation.

[6] Bergmann P, Fauser M, Sattlegger D, Steger C (2019). Mvtec ad — a comprehensive real-world dataset for unsupervised anomaly detection.

[7] Chen M, Radford A, Wu J, Heewoo J, Dhariwal P (2020). Generative pretraining from pixels.

[8] Child R, Gray S, Radford A, Sutskever I (2019). Generating long sequences with sparse transformers.

[9] Choromanski K, Likhosherstov V, Dohan D, Song X, Gane A, Sarlos T, Hawkins P, Davis J, Mohiuddin A, Kaiser L, Belanger D (2020). Rethinking attention with performers.

[10] Clark K, Vendt B, Smith K, Freymann J, Kirby J, Koppel P, Moore S, Phillips S, Maffitt D, Pringle M, Tarbox L (2013). The cancer imaging archive (tcia): Maintaining and operating a public information repository. Journal of Digital Imaging.

[11] Clark K, Vendt B, Smith K, Freymann J, Kirby J, Koppel P, Moore S, Phillips S, Maffitt D, Pringle M, Tarbox L (2013). The cancer imaging archive (tcia): Maintaining and operating a public information repository. Journal of Digital Imaging.

[12] Decuyper M, Maebe J, Holen RV, Vandenberghe S (2021). Artificial intelligence with deep learning in nuclear medicine and radiology. EJNMMI Physics.

[13] Dhariwal P, Jun H, Payne C, Kim JW, Radford A, Sutskever I (2020). Jukebox: A generative model for music.

[14] Dinsdale NK, Bluemke E, Sundaresan V, Jenkinson M, Smith SM, Namburete AI (2022). Challenges for machine learning in clinical translation of big data imaging studies. Neuron.

[15] Dosovitskiy A, Beyer L, Kolesnikov A, Weissenborn D, Zhai X, Unterthiner T, Dehghani M, Minderer M, Heigold G, Gelly S, Uszkoreit J (2020). An image is worth 16x16 words: Transformers for image recognition at scale.

[16] Gatidis S, Hepp T, Früh M, Fougère CL, Nikolaou K, Pfannenberg C, Schölkopf B, Küstner T, Cyran C, Rubin D (2022). A whole-body fdg-pet/ct dataset with manually annotated tumor lesions. Scientific Data.

[17] Gevaert O, Xu J, Hoang CD, Leung AN, Xu Y, Quon A, Rubin DL, Napel S, Plevritis SK (2012). Non–small cell lung cancer: Identifying prognostic imaging biomarkers by leveraging public gene expression microarray data—methods and preliminary results. Radiology.

[18] Graham MS, Tudosiu PD, Wright P, Pinaya WHL, Jean-Marie U, Mah Y, Teo J, Jäger RH, Werring D, Nachev P, Ourselin S (2022). Transformer-based out-of-distribution detection for clinically safe segmentation.

[19] Jurdi RE, Petitjean C, Honeine P, Abdallah F (2021). Coordconv-unet: Investigating coordconv for organ segmentation. IRBM.

[20] Liu R, Lehman J, Molino P, Such FP, Frank E, Sergeev A, Yosinski J (2018). An intriguing failing of convolutional neural networks and the coordconv solution.

[21] van den Oord A, Vinyals O, Kavukcuoglu K (2017). Neural discrete representation learning.

[22] Patel A, Tudosiu PD, Pinaya WHL, Cook G, Goh V, Ourselin S, Cardoso MJ (2022). Cross attention transformers for multi-modal unsupervised whole-body pet anomaly detection.

[23] Pinaya WHL, Tudosiu PD, Gray R, Rees G, Nachev P, Ourselin S, Cardoso MJ (2021). Unsupervised brain anomaly detection and segmentation with transformers.

[24] Schlegl T, Seeböck P, Waldstein SM, Schmidt-Erfurth U, Langs G (2017). Unsupervised anomaly detection with generative adversarial networks to guide marker discovery.

[25] Tudosiu PD, Pinaya WHL, Graham MS, Borges P, Fernandez V, Yang D, Appleyard J, Novati G, Mehra D, Vella M, Nachev P (2022). Morphology-preserving autoregressive 3d generative modelling of the brain.

[26] Valiant LG (1984). A theory of the learnable. Communications of the ACM.

[27] Varoquaux G, Cheplygina V (2022). Machine learning for medical imaging: methodological failures and recommendations for the future. npj Digital Medicine.

[28] Vaswani A, Shazeer N, Parmar N, Uszkoreit J, Jones L, Gomez AN, Kaiser L, Polosukhin I (2017). Attention is all you need.

[29] Wu K, Peng H, Chen M, Fu J, Chao H (2021). Rethinking and improving relative position encoding for vision transformer.

