# Supplementary material for: Geometry-invariant abnormality detection
Source: Med Image Comput Comput Assist Interv. Author manuscript; Available in PMC 2024 Aug 28. (PMC7616404; doi:10.1007/978-3-031-43907-0_29)
Supplement: Appendix [file EMS198178-supplement-Appendix.pdf]

A VQ-VAE Implementation

The VQ-VAE model was trained using a jukebox loss that is given as:

$$L_{VQVAE} = \|(\mathbf{x} - \hat{\mathbf{x}})\|_2^2 + \||STFT(\mathbf{x})| - |STFT(\hat{\mathbf{x}})|\|_2^2 + \beta \|z_e(\mathbf{x}) - sg[\mathbf{e}]\|_2^2 + \|sg[z_e(\mathbf{x})] - \mathbf{e}\|_2^2 \quad (1)$$

where sg stands for a stop gradient operator to stop gradients from flowing back into their argument. The loss used from [13] uses a spectral loss component that is based on the magnitude of the Fourier Transformer of the original and reconstructed image. From equation 1 the first term seen is the L2 pixel loss, whilst the second term represents the spectral loss between the original and reconstruction. Here SFTF stands for the short-time Fourier transform. The third term is the commitment cost to ensure the encoder commits to the codebook. The final term is to move the codebook embedding vectors towards the encoder output. We replace this final term with an exponential moving average update for the codebook as implemented in [21]. During training, a β of 0.25 was used.

The architecture used for the VQ-VAE model used an encoder consisting of three downsampling layers that contain a convolution with stride 2 and kernel size 4 followed by a ReLU activation and 3 residual blocks. Each residual block consists of a kernel of size 3, followed by a ReLU activation, a convolution of kernel size 1 and another ReLU activation. Similar to the encoder, the decoder has 3 layers of 3 residual blocks, each followed by a transposed convolutional layer with stride 2 and kernel size 4. Finally, before the last transposed convolutional layer, a Dropout layer with a probability of 0.05 is added. The VQ-VAE codebook used had 256 atomic elements (vocabulary size), each of length 128. The CT VQ-VAE was identical in hyperparameters except each codebook vector has length 64. To train the VQ-VAE networks, we used an ADAM optimiser with a learning rate of 1e-4 and an exponential learning rate decay with a gamma of 0.9999. Training was run for 1000 epochs with a batch size of 3. During training, the data was augmented with Gaussian noise, contrast adjustment, intensity shifts, translations, rotations, scaling and elastic deformations. For training for the model with ranging geometric profiles, random crops to training images were applied to 60% of images from the whole body datasets with ranging cropping sizes and starting positions.

B Transformer Implementation

Once the VQ-VAE model was trained, and training data could be encoded, a Transformer could then be trained off the encoded images, using their discrete latent representations. The self-attention mechanism is best described as a mapping of intermediate representations of three position-wise linear layers onto three representations denoted by the Value (V), key (K) and query (Q) [28]. With d_k denoting the dimension of the key, query and value vectors, the attention mechanism is calculated as follows:

$$Attn(Q, K, V) = softmax\left(\frac{QK^T}{\sqrt{d_k}}\right)V \quad (2)$$

The Transformer success relies on the self-attention mechanisms employed to capture the interactions between inputs in the sequence regardless of their relative position to one another. This relies on the inner product between elements of the sequence and as such the network scales quadratically with sequence length. This is a key limitation when applied to image data. In this work, we use more scalable Performer variant [9].

The performer used in this work corresponds to a decoder Transformer architecture with 14 layers, each with 8 heads, and an embedding dimension of 256. Similarly the embedding dimension for the CT data and the spatial conditioning data had an embedding dimension of 256 as well. To train the network, we used an ADAM optimiser with a learning rate of 1e-3, an exponential learning rate decay with a gamma of 0.9999 and cross-entry loss. Furthermore the embedding, feed-forward and attention mechanisms within the network all had a dropout probability 0.1.

The data was augmented and fed to the transformer, to avoid extra overfitting to the original training data. Augmentation was the same as applied to the training data during VQ-VAE training. As such, to get the training latent codes for the Transformer, each training image was augmented 3 times (to increase data training size). These augmented images were then encoded via the trained VQ-VAE, or downsampled in the case of the calculated segmentations to then be fed in to the Transformer to train. For the training of our approach with spatial conditioning, again 60% of samples were randomly cropped before being encoded. Their spatial conditioning was saved at this point to be fed into the Transformer. Training was then performed over 80 epochs with a batch-size of 1.

C VQ-VAE visual reconstruction comparisons

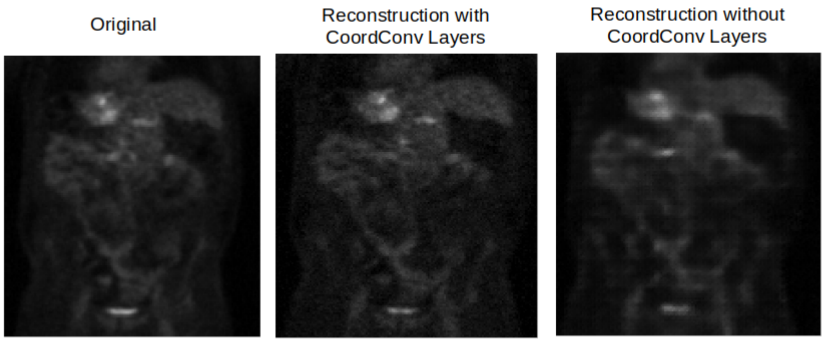

Fig. 5. Reconstruction of PET image (left) trained on data with varying resolution and fields of view with CoordConv layers (middle) and without CoordConv layers (right). Note the blurier reconstruction without CoordConv.
